# Supplementary material for: Decoding brain states on the intrinsic manifold of human brain dynamics across wakefulness and sleep
Source: Commun Biol. 2021 Jul 9;4:854. doi: 10.1038/s42003-021-02369-7 (PMC8270946; doi:10.1038/s42003-021-02369-7)
Supplement: Supplementary file 3 — Description of Additional Supplementary Files [file 42003_2021_2369_MOESM3_ESM.pdf]

Description of Additional Supplementary Files

File name: Supplementary Movie 1

Description: *Temporal evolution of the fMRI BOLD data during wakefulness and sleep embedded in lower dimensional spaces for one subject.* The video shows the intrinsic three-dimensional manifold of subject 7, with color coding for both (A) sleep stage and (B) time-index. fMRI BOLD data shows smooth intra-stage transitions and inter-stage shortcuts. The frame rate is 10 times faster**.**
