# Supplementary material for: Decoding brain states on the intrinsic manifold of human brain dynamics across wakefulness and sleep
Source: Commun Biol. 2021 Jul 9;4:854. doi: 10.1038/s42003-021-02369-7 (PMC8270946; doi:10.1038/s42003-021-02369-7)
Supplement: Supplementary file 5 — Reporting Summary [file 42003_2021_2369_MOESM5_ESM.pdf]

nature research

Corresponding author(s): Joan Rué Queralt

Last updated by author(s): Jul 9, 2020

Reporting Summary

Nature Research wishes to improve the reproducibility of the work that we publish. This form provides structure for consistency and transparency in reporting. For further information on Nature Research policies, see our Editorial Policies and the Editorial Policy Checklist.

Statistics

For all statistical analyses, confirm that the following items are present in the figure legend, table legend, main text, or Methods section.

n/a	Confirmed	
[ ]	[x]	The exact sample size (*n*) for each experimental group/condition, given as a discrete number and unit of measurement
[ ]	[x]	A statement on whether measurements were taken from distinct samples or whether the same sample was measured repeatedly
[ ]	[x]	The statistical test(s) used AND whether they are one- or two-sided<br>*Only common tests should be described solely by name; describe more complex techniques in the Methods section.*
[ ]	[x]	A description of all covariates tested
[ ]	[x]	A description of any assumptions or corrections, such as tests of normality and adjustment for multiple comparisons
[ ]	[x]	A full description of the statistical parameters including central tendency (e.g. means) or other basic estimates (e.g. regression coefficient) AND variation (e.g. standard deviation) or associated estimates of uncertainty (e.g. confidence intervals)
[ ]	[x]	For null hypothesis testing, the test statistic (e.g. *F*, *t*, *r*) with confidence intervals, effect sizes, degrees of freedom and *P* value noted<br>*Give P values as exact values whenever suitable.*
[x]	[ ]	For Bayesian analysis, information on the choice of priors and Markov chain Monte Carlo settings
[x]	[ ]	For hierarchical and complex designs, identification of the appropriate level for tests and full reporting of outcomes
[ ]	[x]	Estimates of effect sizes (e.g. Cohen's *d*, Pearson's *r*), indicating how they were calculated

Our web collection on statistics for biologists contains articles on many of the points above.

Software and code

Policy information about availability of computer code

Data collection	No software was used for data collection.
Data analysis	MRI preprocessing implemented in Vision Analyzer2 (Brain Products, Germany), fMRI preprocessing using Statistical Parametric Mapping (SPM8) and custom matlab scripts. Data analysis was performed with custom matlab scripts that are available from the corresponding author upon reasonable request.

For manuscripts utilizing custom algorithms or software that are central to the research but not yet described in published literature, software must be made available to editors and reviewers. We strongly encourage code deposition in a community repository (e.g. GitHub). See the Nature Research guidelines for submitting code & software for further information.

Data

Policy information about availability of data

All manuscripts must include a data availability statement. This statement should provide the following information, where applicable:

- Accession codes, unique identifiers, or web links for publicly available datasets
- A list of figures that have associated raw data
- A description of any restrictions on data availability

All data generated in this study are available from the corresponding author upon reasonable request

Field-specific reporting

Please select the one below that is the best fit for your research. If you are not sure, read the appropriate sections before making your selection.

- [x] Life sciences
- [ ] Behavioural & social sciences
- [ ] Ecological, evolutionary & environmental sciences

For a reference copy of the document with all sections, see nature.com/documents/nr-reporting-summary-flat.pdf

Life sciences study design

All studies must disclose on these points even when the disclosure is negative.

Sample size	The sample size (18 subjects) was chosen based on the literature of studies with similar purpose of demonstrating the applicability of a newly proposed technique.
Data exclusions	The subset chosen here was selected based on the condition of spending a minimum of 3 minutes (87 TRs) in each of the three non-REM sleep stages considered here (i.e. N1, N2, N3), as well as achieving successful EEG, fMRI, and physiological data recording and quality. Inclusion conditions were set to get the maximal representative sampling of each stage, while still maintaining a significant number of participants.
Replication	To test how well the classification generalizes to independent datasets, we used cross-validation: we splitted the data into different subsets which were used exclusively either in the training or testing phase of the cross-validation.
Randomization	Randomization was only used to create surrogate time series to test whether the results obtained were due to preprocessing artifacts. For this reason, we applied the same pipeline to time series of each participant that were temporally shuffled before being processed. Randomization in Monte Carlo (computer) simulations were performed using uniformly distributed randomization method available in MATLAB
Blinding	All participants where considered to be part of the same healthy population. The only group labels were sleeping stages. Data acquisition and analysis was performed without blinding process given that this was an exploratory study, and there were no a priori hypothesis.

Reporting for specific materials, systems and methods

We require information from authors about some types of materials, experimental systems and methods used in many studies. Here, indicate whether each material, system or method listed is relevant to your study. If you are not sure if a list item applies to your research, read the appropriate section before selecting a response.

Materials & experimental systems

n/a	Involved in the study	
☐	☐	Antibodies
☐	☐	Eukaryotic cell lines
☐	☐	Palaeontology and archaeology
☐	☐	Animals and other organisms
☐	☒	Human research participants
☐	☐	Clinical data
☐	☐	Dual use research of concern

Methods

n/a	Involved in the study	
☐	☐	ChIP-seq
☐	☐	Flow cytometry
☐	☒	MRI-based neuroimaging

Antibodies

Antibodies used	n/a
Validation	n/a

Eukaryotic cell lines

Policy information about cell lines

Cell line source(s)	n/a
Authentication	n/a
Mycoplasma contamination	n/a
Commonly misidentified lines (See ICLAC register)	n/a

Palaeontology and Archaeology

Specimen provenance	n/a
Specimen deposition	n/a
Dating methods	n/a

☐ Tick this box to confirm that the raw and calibrated dates are available in the paper or in Supplementary Information.

Ethics oversight	n/a

Note that full information on the approval of the study protocol must also be provided in the manuscript.

Animals and other organisms

Policy information about studies involving animals; ARRIVE guidelines recommended for reporting animal research

Laboratory animals	n/a
Wild animals	n/a
Field-collected samples	n/a
Ethics oversight	n/a

Note that full information on the approval of the study protocol must also be provided in the manuscript.

Human research participants

Policy information about studies involving human research participants

Population characteristics	The dataset utilized in this study is a subset of a sleep dataset, consisting of 58 subjects scanned for 52 minutes with simultaneous EEG-fMRI, containing subjects who exhibit wakefulness as well as sleep during the scanning session (23% N1, 19% N2, and 10% N3 sleep, age 23.5±3.3, 39 females). Reference to original dataset: Tagliazucchi, E. & Laufs, H. Decoding wakefulness levels from typical fMRI resting-state data reveals reliable drifts between wakefulness and sleep. Neuron. 82(3), 695–708 (2014).
Recruitment	The recruitment was done following the ethics approved by the Ethics Committee of the Goethe University of Frankfurt am Main, Germany, written informed consent, participants were reimbursed for their participation.
Ethics oversight	Ethics Committee of the Goethe University of Frankfurt am Main, Germany

Note that full information on the approval of the study protocol must also be provided in the manuscript.

Clinical data

Policy information about clinical studies

All manuscripts should comply with the ICMJEguidelines for publication of clinical research and a completedCONSORT checklist must be included with all submissions.

Clinical trial registration	n/a
Study protocol	n/a
Data collection	n/a
Outcomes	n/a

Dual use research of concern

Policy information about dual use research of concern

Hazards

Could the accidental, deliberate or reckless misuse of agents or technologies generated in the work, or the application of information presented in the manuscript, pose a threat to:

No	Yes	
☒	☐	Public health
☒	☐	National security
☒	☐	Crops and/or livestock
☒	☐	Ecosystems
☒	☐	Any other significant area

Experiments of concern

Does the work involve any of these experiments of concern:

No	Yes	
☒	☐	Demonstrate how to render a vaccine ineffective
☒	☐	Confer resistance to therapeutically useful antibiotics or antiviral agents
☒	☐	Enhance the virulence of a pathogen or render a nonpathogen virulent
☒	☐	Increase transmissibility of a pathogen
☒	☐	Alter the host range of a pathogen
☒	☐	Enable evasion of diagnostic/detection modalities
☒	☐	Enable the weaponization of a biological agent or toxin
☒	☐	Any other potentially harmful combination of experiments and agents

ChIP-seq

Data deposition

☐ Confirm that both raw and final processed data have been deposited in a public database such as GEO.

☐ Confirm that you have deposited or provided access to graph files (e.g. BED files) for the called peaks.

Data access links<br>*May remain private before publication.*	n/a
Files in database submission	n/a
Genome browser session<br>(e.g. UCSC)	n/a

Methodology

Replicates	n/a
Sequencing depth	n/a
Antibodies	n/a
Peak calling parameters	n/a
Data quality	n/a
Software	n/a

Flow Cytometry

Plots

Confirm that:

☐ The axis labels state the marker and fluorochrome used (e.g. CD4-FITC).

☐ The axis scales are clearly visible. Include numbers along axes only for bottom left plot of group (a 'group' is an analysis of identical markers).

☐ All plots are contour plots with outliers or pseudocolor plots.

☐ A numerical value for number of cells or percentage (with statistics) is provided.

Methodology

Sample preparation	n/a
Instrument	n/a
Software	n/a
Cell population abundance	n/a
Gating strategy	n/a

☐ Tick this box to confirm that a figure exemplifying the gating strategy is provided in the Supplementary Information.

Magnetic resonance imaging

Experimental design

Design type	Resting state
Design specifications	Participants laid in the scanner during an average time length of 52 minutes, after being asked not to fight sleep.
Behavioral performance measures	Sleep scoring mesured with an optimized polysomnographic setting (chin and tibial EMG, ECG, EOG recorded bipolarly [sampling rate 5 kHz, low pass filter 1 kHz], 30 EEG channels recorded with FCz as the reference [sampling rate 5 kHz, low pass filter 250 Hz], and pulse oximetry, respiration recorded via sensors from the Trio [sampling rate 50 Hz]) and MR scanner compatible devices (BrainAmp MR+, BrainAmp ExG; Brain Products, Gilching, Germany).

Acquisition

Imaging type(s)	functional and structural
Field strength	3T
Sequence & imaging parameters	1505 volumes of T2*-weighted echo-planar images, TR/TE=2080 ms/30 ms, matrix 64×64, voxel size 3×3×2 mm3, distance factor 50%; FOV 192 mm2
Area of acquisition	Whole brain

Diffusion MRI ☐ Used ☒ Not used

Preprocessing

Preprocessing software	MRI and pulse artifact correction were performed based on the average artifact subtraction (AAS) method 43 as implemented in Vision Analyzer2 (Brain Products, Germany).
Normalization	Using Statistical Parametric Mapping (SPM8) EPI data were realigned, normalized (MNI space)
Normalization template	MNI space
Noise and artifact removal	Using Statistical Parametric Mapping (SPM8) EPI data were spatially smoothed (Gaussian kernel, 8 mm3 full width at half maximum). Data were band-pass filtered in the range 0.01–0.1 Hz using a sixth-order Butterworth filter.
Volume censoring	n/a

Statistical modeling & inference

Model type and settings	Supervised classification (Support vector machine) after both linear dimensionality reduction (PCA) and nonlinear dimensionality reduction (Laplacian Eigenmaps).
Effect(s) tested	Accuracy of classification of time points belonging to different sleep stages after dimensionality reduction.

Specify type of analysis: ☐ Whole brain ☒ ROI-based ☐ Both

Anatomical location(s): Automated Anatomical Labeling with 90 regions of interest (AAL-90).

Statistic type for inference (See Eklund et al. 2016)	We use time point-wise classification based on the localization of the time point in the intrinsic manifold (or PCA space).
Correction	Yes, we corrected for multiple comparisons using false discovery rate correction method.

Models & analysis

n/a	Involved in the study
☐	☒ Functional and/or effective connectivity
☐	☒ Graph analysis
☐	☒ Multivariate modeling or predictive analysis

Functional and/or effective connectivity	We used the Coherence Connectivity Dynamics matrix, which provides information about the time-dependency of spatial phase coupling dynamics.
Graph analysis	In this study, graphs were constructed in the time domain, with their nodes being the time points, and the edges where established using a Relaxed Minimum Spanning Tree algorithm on the Coherence Connectivity Dynamics.
Multivariate modeling and predictive analysis	We first performed dimensionality reduction using Laplacian Eigenmaps, to get relevant features from the Coherence Connectivity Dynamics. We then performed classification on these feature space, using Support Vector Machines to classify time points belonging to one class (sleep stage) or another. All classifications were performed with only 2 classes at a time.
